# Analysis of Long COVID characteristics and risk factors in individuals infected with COVID-19: a follow-up study based on a cohort of 2,792 participants

**DOI:** 10.3389/fpubh.2026.1760355

**Published:** 2026-03-10

**Authors:** Xiujie Chu, Sai Hou, Qian Zhu, Jingru Chang, Lei Gong, Jiabing Wu

**Affiliations:** 1 Institute for the Prevention and Control of Infectious Diseases, Anhui Provincial Center for Disease Control and Prevention, Hefei, Anhui, China; 2 Health Emergency Center, Anhui Provincial Center for Disease Control and Prevention, Hefei, Anhui, China

**Keywords:** biomarker, characteristic, cohort, Long COVID, risk factor

## Abstract

**Background:**

Since the emergence of SARS-CoV-2 in 2019, Long COVID has emerged as a significant global public health challenge. The identification of accessible biomarkers and risk factors is critical to enabling early intervention and improving long-term outcomes.

**Methods:**

This prospective cohort study enrolled 2,792 individuals with confirmed COVID-19 from Anhui Province in September 2024. A propensity score matching analysis was performed using a 1:4 ratio. Cases and matched controls were selected from cohort, serum sample were analyzed to assess hematological parameters. Multivariable logistic regression models were applied to identify independent risk factors associated with the development of Long COVID.

**Results:**

2,792 participants (average age 51.64 years) identified 182 (6.52%) long COVID patients during follow-up. Common symptoms included fatigue, cough, insomnia, throat discomfort, and appetite loss. After propensity score matching, risk factors were age, more severe acute symptoms. Long COVID patients exhibited higher red blood cell counts but lower hemoglobin-related indices and platelet count.

**Conclusion:**

This study confirms the persistent risk of Long COVID following reinfection, with heightened susceptibility associated with advanced age, specific acute-phase symptoms. Alterations in routine hematological parameters may serve as valuable biomarkers for the monitoring and management of Long COVID.

## 1 Background

Since the initial report of the Coronavirus Disease 2019 (COVID-19) in 2019, the virus has spread rapidly across the globe, infecting approximately 779 million people and causing an estimated 7.1 million deaths (1). As the pandemic has progressed, the long-term health effects of COVID-19—commonly known as “Long COVID”—have become a central focus of public health research. The term “Long COVID” was first introduced by patient groups to describe persistent symptoms and aftereffects following acute COVID-19 (2). According to the World Health Organization (WHO), Long COVID is defined as a condition characterized by symptoms lasting at least 2 months, occurring within 3 months of confirmed or probable SARS-CoV-2 infection, and not explained by alternative diagnoses (3).

Long COVID has emerged as a significant public health burden amid the surge in SARS-CoV-2 infections worldwide. A comprehensive analysis estimates that the global prevalence of long COVID reaches as high as 45 percent, indicating that more than one-third of the hundreds of millions of individuals recovered from acute COVID-19 may experience persistent health complications (4). Moreover, identifying high-risk populations for long COVID is essential for enabling early intervention and optimizing resource allocation. Although existing studies have identified several potential risk factors, inconsistencies in findings underscore the complexity of research in this area. An extensive cohort study involving over 4,000 COVID-19 survivors reported that advanced age (over 70 years), female sex, presence of more than five symptoms during the first week of acute infection, and pre-existing comorbidities were significantly associated with increased risk of developing long COVID (5). In contrast, a separate large-scale analysis based on electronic health records from approximately 2.1 million patients yielded partially conflicting results. After adjusting for multiple covariates, this study found that the risk of long COVID increased progressively with age (6). Meanwhile, disease severity during the acute phase is broadly recognized as a key predictor, with patients requiring intensive care exhibiting a higher likelihood of developing long COVID (7). These findings offer a preliminary foundation for clinical risk assessment; however, larger-scale prospective studies are still required to validate risk factors.

Currently, given the significant impact of long COVID on population health and the limited understanding of its underlying pathogenic mechanisms, there is an urgent need to identify cost-effective biomarkers for monitoring and diagnosing long COVID symptoms, particularly through widely accessible and routinely collected samples. It's worth noting that most prior long COVID cohort studies have focused on epidemiological characteristics, symptom profiles and risk factor identification, yet few prospectively explored routine hematology parameters as biomarkers for long COVID in China. A 1-year follow-up study of 214 infected individuals revealed that iron metabolism disorders during the acute phase were significantly associated with the subsequent development of long COVID (8). Routine blood tests and assessments of liver and kidney function, which are economical, widely available, and operationally efficient, can provide clinically meaningful insights for the surveillance and diagnosis of long COVID. However, there remains a notable lack of systematic, long-term studies that explore the evolution of these serum markers in long COVID patients and examine their associations with specific symptom clusters. To address this gap, the present study established a Long-COVID symptom cohort to investigate factors influencing long COVID manifestations and to characterize changes in relevant serum biomarkers.

## 2 Materials and methods

### 2.1 Research subjects

In September 2024, Anhui Province experienced a peak in reported notifiable COVID-19 cases through the surveillance system. Subsequently, we initiated this investigation. This study employed a prospective cohort design and enrolled COVID-19 patients from Anhui Province in September 2024. Six Cities with the highest incidence rates during the period were selected as study sites. On September 20, 2024, the research team conducted the first systematic training session for epidemiological investigators via video conference. The baseline assessment was conducted during the same month, with a subsequent follow-up for long COVID symptoms carried out in December 2024. Inclusion criteria were: (1) permanent residency in the local area and willingness to participate in follow-up; (2) age over 18 years, encompassing all adult age groups and both sexes. Exclusion criteria included refusal to participate or death prior to the survey. This study has been approved by the Medical Research Ethics Committee of Anhui Provincial Center for Disease Control and Prevention (SL-2024-73003-01).

### 2.2 Investigation methods

Eligible COVID-19 patients were identified through the National Notifiable Disease Reporting System (NNDRS) of China, NNDRS is a real-time infectious disease surveillance system led by the National Health Commission (NHC) and constructed by the Chinese Center for Disease Control and Prevention, Built on a five-level network spanning from township-level to national-level health institutions and a three-level platform covering prefecture, provincial and national tiers, the system has been rolled out across 168,000 medical and health institutions nationwide, monitoring 40 categories of notifiable infectious diseases. They were subsequently enrolled in a follow-up cohort to monitor the progression of infection. First, CDC personnel involved in field investigations received centralized training. Meanwhile, data on age, gender, vaccination status, and infection severity were extracted directly from the surveillance system. Clinical classification was based on the 10th Edition of the Diagnosis and Treatment Protocol for Novel Coronavirus Infection (9), categorizing cases into non-hospitalized, mild, moderate, severe, and critical types. After obtaining the informed consent of the participants interviews were conducted using a standardized baseline questionnaire. Trained CDC professionals conducted face-to-face or telephone interviews to complete the baseline questionnaire (see Supplemental instrument 1). This questionnaire captured core information including participants' demographic characteristics, initial symptoms and healthcare-seeking history related to COVID-19 infection, daily lifestyle behaviors, and prior history of underlying diseases. Three months after the completion of the baseline survey, the research team organized a second specialized training session for investigators, immediately followed by the initiation of the follow-up survey. A customized follow-up questionnaire (see Supplemental instrument 2) was administered during this phase, focusing on collecting updated basic personal information and data on the occurrence and progression of long-term symptoms following COVID-19 infection. Upon completion of the follow-up survey, the Anhui Provincial Center for Disease Control and Prevention was responsible for the centralized collection, verification, and aggregation of all questionnaire data. Long COVID is a chronic condition defined by the World Health Organization (WHO). The condition was defined as persistent or new-onset symptoms that last for at least 2 months following initial SARS-CoV-2 infection and cannot be explained by alternative diagnoses. Determine whether an individual meets the criteria for long COVID by responding to the following three questions.

1. Since the last survey, do you still have symptoms (specifically those that may be caused by COVID-19), and the duration is more than two months? If no, skip to the end.
2. Did these symptoms newly appear after being infected with COVID-19?
3. Do you think these symptoms are more severe than before this COVID-19 infection?

Based on these follow-up findings, a case-control study was conducted. The research team contacted all confirmed cases to inquire about their willingness to participate in subsequent hematological tests. For cases who voluntarily consented to the tests, the frequency matching method was adopted to select non-post-COVID-19 condition individuals matched by age and gender from the same study sites as the control group. With informed consent obtained from all participants, serum samples were collected for subsequent analyses, including complete blood count, liver function, and kidney function tests.

### 2.3 Laboratory tests

Fasting venous blood samples were collected from subjects in the morning. Each sample was immediately labeled, documented for sample information, and placed in ice boxes to maintain cold chain integrity. Two tubes of whole blood were collected from each participant: one anticoagulant tube containing EDTA (2–3 ml) for complete blood count analysis, and one non-anticoagulant tube (2–3 ml) for liver and kidney function testing. Blood routine and biochemical tests were performed promptly after sample collection. Serum analyses were conducted using an automated biochemical analyzer.

### 2.5 Statistical methods

R software (version 4.1.2) was used for statistical analysis. This study conducted a normality test using the Kolmogorov-Smirnov test. Clinical characteristics were systematically compared between patients with long COVID and those without long COVID using the chi-square test for categorical variables and the *Z*-test for continuous variables. Multicollinearity was assessed via variance inflation factor (VIF). Multivariate binary logistic regression model was applied to adjust for potential confounding factors and to identify variables significantly associated with the presence of long COVID. Variable selection is conducted using the two-way stepwise regression analysis method. Propensity score matching (PSM) was conducted at a 4:1 ratio (long COVID: non-long COVID), incorporating key baseline covariates—including gender, BMI, living habit (including smoking status, alcohol consumption, exercise situation, and sleep situation), number of infection, number of vaccinations and chronic disease—to ensure balanced group distribution and reduce selection bias. A univariate comparative analysis was subsequently performed on the matched cohort to assess differences in clinical outcomes between the two groups rigorously. To maximize sample retention while maintaining robust comparability, nearest neighbor matching was implemented with a caliper width of 0.2, and balance across groups was formally evaluated using standardized mean differences (SMD), with SMD < 0.1 indicating adequate balance.

## 3 Survey results

### 3.1 Basic information

**Figure 1:**
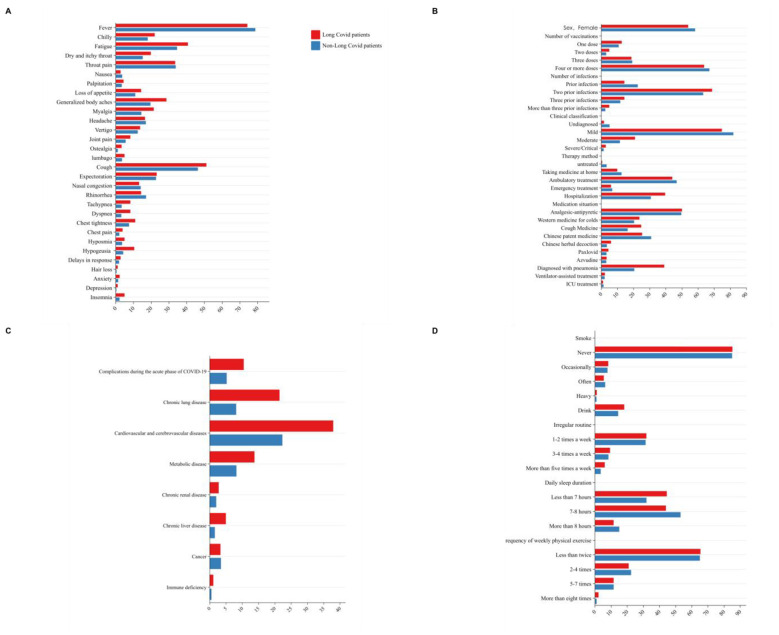
The basic characteristics of individuals with long COVID and those without long COVID after infection. **(A)** Acute phase symptoms. **(B)** Epidemiological characteristics and clinical features. **(C)** Characteristics of underlying diseases and comorbidities. **(D)** living habit.

Patients diagnosed with COVID-19 in Anhui Province in September were selected based on the notifiable infectious disease reporting system. Initially, a total of 3,120 participants were included in the baseline survey. Subsequently, after excluding the questionnaires that were lost to follow-up, duplicated, or conflicting, a total of 2,792 questionnaires were finally included. (See Supplementary Figure 1). The mean age was 51.64 ± 20.97 years, the mean height was 165 ± 7.77 cm, and the mean weight was 62.17 ± 11.48 kg. Of the participants, 1,176 (42.1%) were maleand 1,616 were female (57.9%). All had received at least one dose of a SARS-CoV-2 vaccine (100%), with the majority having received three or more doses (*n* = 2,399, 85.9%). A total of 77.9% (*n* = 2,176) reported two or more prior SARS-CoV-2 infections. Most cases were classified as mild (*n* = 2,272, 81.4%). Regarding treatment, 87 (3.1%) received no medical intervention, 345 (12.4%) were managed with home medication, 1,487 (53.3%) were treated in outpatient or emergency departments, and 873 (31.3%) were hospitalized. See Figure 1.

In terms of lifestyle factors, 419 (15.0%) were current smokers, 408 (14.6%) consumed alcohol, 1,576 (56.5%) maintained regular daily routines, and the majority (*n* = 1,464, 52.4%) slept 7–8 h per day. The most frequently reported symptoms during the acute phase of infection were fever (*n* = 2,188, 78.4%), cough (*n* = 1,302, 46.6%), fatigue (*n* = 976, 35.0%), throat pain (*n* = 944, 33.8%), and expectoration (*n* = 635, 22.7%). Less common symptoms included depression (*n* = 11, 0.4%), alopecia (*n* = 14, 0.5%), and ostealgia (*n* = 37, 1.3%). Notably, only 50 individuals (1.79%) were completely asymptomatic. The most commonly used treatments included single-agent antipyretic and analgesic drugs such as ibuprofen and acetaminophen (*n* = 1,385, 49.6%) and traditional Chinese patent medicines such as Lianhua Qingwen (*n* = 852, 30.5%). A total of 601 participants (21.7%) were diagnosed with pneumonia via chest CT, but only 58 (2.1%) required mechanical ventilation, and 38 (1.4%) who received intensive care were admitted to the ICU. In terms of complications, the main comorbidities reported by respondents included cardiovascular and cerebrovascular diseases (*n* = 651, 23.3%), metabolic disorders (*n* = 251, 9.0%), and endocrine diseases (*n* = 219, 8.6%). See Figure 1.

### 3.2 Follow-up results of the investigation

At the 3-month follow-up after symptom onset, 218 individuals (7.8%) were found to have persistent symptoms since their last assessment. Of these, 168 (6.0%) reported that the symptoms were new-onset following SARS-CoV-2 infection, while among the remaining 50, 14 indicated a worsening of pre-existing conditions compared to baseline. According to established diagnostic criteria for long COVID, a total of 182 patients (6.5%) met the case definition and were classified as having long COVID.

We combined the original height and weight variables into a BMI variable. The VIF results indicate all VIF values < 3 indicating no significant collinearity. Among individuals reporting only one SARS-CoV-2 infection, the prevalence of long-term symptoms was lower, with a long COVID rate of 4.2% (26/616). In contrast, among those who reported two SARS-CoV-2 infection, the prevalence was 7.5% (121/1796), and among those who reported three SARS-CoV-2 infection, it reached 7.8% (26/333). The most commonly reported persistent symptoms at the 3-month follow-up included fatigue (*n* = 74, 40.7%), cough (*n* = 40, 22.0%), insomnia (*n* = 34, 18.7%), Dry and itchy throat (*n* = 32, 17.6%), and loss of appetite (*n* = 30, 16.5%). See Table 1.

**Table 1:** The basic characteristics and clinical features table in participant.

**Variables**	**Non long COVID (*n* = 2,610)**	**Long COVID (*n* = 182)**	**Statistic**	***P***
Age, M (Q1, Q3)	52.00 (31.00, 70.00)	59.00 (44.75, 72.00)	*Z* = −4.61	**< 0.001**
BMI, M (Q1, Q3)	22.49 (20.31, 24.69)	23.12 (21.02, 24.91)	*Z* = −2.05	**0.041**
**Gender**, ***n*** **(%)**			χ^2^ = 1.30	0.254
Male	1,092 (41.84)	84 (46.15)		
Female	1518 (58.16)	98 (53.85)		
**Age group**, ***n*** **(%)**			χ^2^ = 26.37	**< 0.001**
18–39	1,009 (38.66)	38 (20.88)		
40–59	591 (22.64)	55 (30.22)		
60–79	736 (28.20)	72 (39.56)		
≥80	274 (10.50)	17 (9.34)		
**Number of vaccine**, ***n*** **(%)**			χ^2^ = 2.80	0.423
One dose	282 (10.80)	23 (12.64)		
Two doses	79 (3.03)	9 (4.95)		
Three doses	500 (19.16)	34 (18.68)		
Four or more doses	1,749 (67.01)	116 (63.74)		
**Number of infection**, ***n*** **(%)**			χ^2^ = 10.41	**0.015**
Prior infection	590 (22.61)	26 (14.29)		
Two prior infections	1,648 (63.14)	121 (66.48)		
Three prior infections	307 (11.76)	26 (14.29)		
More than three prior infections	65 (2.49)	9 (4.95)		
**Clinical classification**, ***n*** **(%)**			χ^2^ = 19.21	**< 0.001**
Undiagnosed	134 (5.13)	3 (1.65)		
Mild	2,136 (81.84)	136 (74.73)		
Moderate	301 (11.53)	38 (20.88)		
Severe/critical	39 (1.49)	5 (2.75)		
**Therapy method**, ***n*** **(%)**			χ^2^ = 9.77	**0.044**
Untreated	86 (3.30)	1 (0.55)		
Taking medicine at home	327 (12.53)	18 (9.89)		
Ambulatory treatment	1,219 (46.70)	80 (43.96)		
Emergency treatment	177 (6.78)	11 (6.04)		
Hospitalization	801 (30.69)	72 (39.56)		
**Analgesic antipyretic**, ***n*** **(%)**			χ^2^ = 0.01	0.912
No	1,316 (50.42)	91 (50.00)		
Yes	1,294 (49.58)	91 (50.00)		
**Western medicine for colds**, ***n*** **(%)**			χ^2^ = 1.15	0.284
No	2,080 (79.69)	139 (76.37)		
Yes	530 (20.31)	43 (23.63)		
**Cough medicine**, ***n*** **(%)**			χ^2^ = 8.57	**0.003**
No	2,184 (83.68)	137 (75.27)		
Yes	426 (16.32)	45 (24.73)		
**Chinese patent medicine**, ***n*** **(%)**			χ^2^ = 2.52	0.112
No	1,804 (69.12)	136 (74.73)		
Yes	806 (30.88)	46 (25.27)		
**Chinese herbal decoction**, ***n*** **(%)**			χ^2^ = 3.29	0.070
No	2,520 (96.55)	171 (93.96)		
Yes	90 (3.45)	11 (6.04)		
**Paxlovid**, ***n*** **(%)**			χ^2^ = 0.74	0.390
No	2,526 (96.78)	174 (95.60)		
Yes	84 (3.22)	8 (4.40)		
**Azvudine**, ***n*** **(%)**			χ^2^ = 0.03	0.861
No	2,530 (96.93)	176 (96.70)		
Yes	80 (3.07)	6 (3.30)		
**Diagnosed with pneumonia**, ***n*** **(%)**			χ^2^ = 34.50	**< 0.001**
No	2,076 (79.54)	111 (60.99)		
Yes	534 (20.46)	71 (39.01)		
**Received ventilator assisted treatment**, ***n*** **(%)**			χ^2^ = 0.00	1.000
No	2,556 (97.93)	178 (97.80)		
Yes	54 (2.07)	4 (2.20)		
**Be admitted to the ICU for treatment**, ***n*** **(%)**			χ^2^ = 0.00	1.000
No	2,574 (98.62)	180 (98.90)		
Yes	36 (1.38)	2 (1.10)		
**Complications during the acute phase of COVID-19**, ***n*** **(%)**			χ^2^ = 8.87	**0.003**
No	2,474 (94.79)	163 (89.56)		
Yes	136 (5.21)	19 (10.44)		

Z: Mann-Whitney test, χ^2^, Chi-square test; -, Fisher exact; M, Median; Q1, 1st Quartile; Q3, 3st Quartile.

### 3.3 Multivariate analysis of Long COVID risk factors

Univariate analysis demonstrated that the long COVID group had a significantly higher age and BMI compared with the non-long COVID group, and the proportion of participants with more than one infection was also markedly higher in the long COVID group. Furthermore, a significantly higher proportion of individuals in the long COVID group presented with acute-phase symptoms including generalized body aches, fatigue, myalgia, ostealgia, tachypnea, dyspnea, insomnia, and hypogeusia. In addition, long COVID patients were more prone to be diagnosed with pneumonia, to have used common cough medications (e.g., compound glycyrrhiza tablets, ambroxol hydrochloride) during the acute phase, and to suffer from comorbidities including other acute-phase complications, chronic lung disease, metabolic disease, cardiovascular, and cerebrovascular diseases as well as chronic liver disease. See Table 1 and Supplementary Table 1.

Multivariate logistic regression analysis indicated that, compared with patients with a single COVID-19 infection, those with two, three, and more than three infections had a significantly elevated risk of developing Long COVID, with the odds ratio (OR) of 2.01 [95% confidence interval (CI): 1.29–3.13, *P* = 0.002], 2.19 (95% CI: 1.23–3.91, *P* = 0.008), and 3.04 (95% CI: 1.33–6.95, *P* = 0.008), respectively. Hypogeusia during the acute phase of COVID-19 was also found to have a significant independent association with Long COVID (OR = 2.19, 95% CI: 1.26–3.80, *P* = 0.005). A diagnosis of pneumonia in the acute phase was associated with a 1.93-fold increased risk of Long COVID relative to those without a pneumonia diagnosis (OR = 1.93, 95% CI: 1.37–2.72, *P* < 0.001). With respect to age stratification, compared with the 18–39 years age group, the 40–59 years and 60–79 years age groups had a higher risk of developing Long COVID, with ORs of 1.92 (95% CI: 1.23–3.00, *P* = 0.004) and 1.59 (95% CI: 1.01–2.54, *P* = 0.049), respectively. Furthermore, a higher number of pre-existing chronic diseases was significantly correlated with an increased risk of Long COVID (OR = 1.36, 95% CI: 1.15–1.63, *P* < 0.001). See Table 2.

**Table 2:** Multivariate logistic regression analysis of long COVID.

**Variables**	**β**	**S.E**	***Z***	***P***	**OR (95% CI)**
Intercept	−3.91	0.30	−13.00	**< 0.001**	0.02 (0.01–0.04)
**Number infection**					
Prior infection					1.00 (Reference)
Two prior infections	0.70	0.23	3.08	**0.002**	2.01 (1.29–3.13)
Three prior infections	0.79	0.30	2.66	**0.008**	2.19 (1.23–3.91)
More than three prior infections	1.11	0.42	2.64	**0.008**	3.04 (1.33–6.95)
**Generalized body aches**					
No					1.00 (Reference)
Yes	0.30	0.18	1.61	0.106	1.34 (0.94–1.93)
**Hypogeusia**					
No					1.00 (Reference)
Yes	0.78	0.28	2.79	**0.005**	2.19 (1.26–3.80)
**Diagnosed with pneumonia**					
No					1.00 (Reference)
Yes	0.66	0.18	3.73	**< 0.001**	1.93 (1.37–2.72)
**Sleep time**					
Less than 7 hours					1.00 (Reference)
7–8 hours	−0.27	0.17	−1.54	0.123	0.77 (0.55–1.07)
More than 8 hours	−0.45	0.26	−1.73	0.083	0.64 (0.38–1.06)
**Age group**					
18–39					1.00 (Reference)
40–59	0.65	0.23	2.86	**0.004**	1.92 (1.23–3.00)
60–79	0.47	0.24	1.97	**0.049**	1.59 (1.01–2.54)
≥80	−0.02	0.33	−0.05	0.961	0.98 (0.52–1.88)
Chronic disease	0.31	0.09	3.48	**< 0.001**	1.36 (1.15–1.63)

OR, Odds ratio; CI, Confidence interval.

### 3.3 Analysis of risk factors of Long COVID after PSM

A 1:4 propensity score matching (PSM) was performed to balance key baseline covariates, including gender, BMI, living habit (including smoking status, alcohol consumption, exercise situation, and sleep situation), number of infection, number of vaccinations and chronic disease. The matched cohort comprised 889 individuals, including 179 patients (20.1%) with long COVID and 710 (79.9%) without long COVID, see Supplementary Table 2. Statistically significant differences were observed between the two groups in terms of age, age group, generalized body aches, hypogeusia, clinical classification, and pneumonia diagnosis during the acute phase (all *P* < 0.05). Univariate analysis revealed that the long COVID group had a significantly higher mean age than the non-long COVID group. In addition, a markedly higher proportion of patients in the long COVID group presented with generalized body aches and hypogeusia during the acute phase of COVID-19, and these patients were also more likely to be diagnosed with pneumonia in the acute phase. See Supplementary Table 3.

To further examine factors independently associated with long COVID, multivariable logistic regression analysis was conducted. Variables with *p*-values < 0.05 in univariate analyses were retained for inclusion in the multivariable model. Results of the multivariate logistic regression analysis demonstrated that acute-phase hypogeusia was an independent and significant risk factor for long COVID (OR = 2.46, 95% CI: 1.30–4.64, *P* = 0.005). A diagnosis of pneumonia in the acute phase was associated with a 1.86-fold elevated risk of developing long COVID compared with those without acute-phase pneumonia (OR = 1.86, 95% CI: 1.28–2.71, *P* = 0.001). For age stratification, compared with the 18–39 years age group, the 40–59 years and 60–79 years age groups had a significantly increased risk of long COVID, with the corresponding odds ratios of 1.82 (95% CI: 1.14–2.93, *P* = 0.013) and 1.60 (95% CI: 1.01–2.53, *P* = 0.045), respectively. See Table 3.

**Table 3:** Multivariate logistic regression analysis of long COVID after score matching.

**Variables**	**β**	**S.E**	***Z***	***P***	**OR (95% CI)**
Intercept	−2.01	0.19	−10.87	**< 0.001**	0.13 (0.09–0.19)
**Generalized body aches**					
No					1.00 (Reference)
Yes	0.32	0.20	1.58	0.114	1.38 (0.93–2.04)
**Hypogeusia**					
No					1.00 (Reference)
Yes	0.90	0.32	2.78	**0.005**	2.46 (1.30–4.64)
**Diagnosed with pneumonia**					
No					1.00 (Reference)
Yes	0.62	0.19	3.25	**0.001**	1.86 (1.28–2.71)
**Age group**					
18–39					1.00 (Reference)
40–59	0.60	0.24	2.49	**0.013**	1.82 (1.14–2.93)
60–79	0.47	0.23	2.00	**0.045**	1.60 (1.01–2.53)
≥80	−0.09	0.34	−0.28	0.780	0.91 (0.47–1.77)

OR, Odds ratio; CI, Confidence interval.

### 3.4 Long COVID patients may exhibit alterations in anemia-related laboratory parameters.

Three months after COVID-19 infection, we followed up and collected serum samples from 105 long COVID patients and 105 control groups for testing. Among them, the long COVID group had an average age of 55.27 ± years, 54 males (51.4%), 51 females (48.6%), and an average height of 165.82 ± cm. The average weight was 62.43 ± kg. The non-long COVID group had an average age of 53.09 ± years, 54 males (51.4%) and 51 females (48.6%), an average height of 165.72 ± cm, and an average weight of 64.05 ± kg. See Table 4.

**Table 4:** Epidemiological and clinical characteristics of patients with COVID-19 undergoing hematological examinations.

**Variables**	**Non long COVID (*n* = 105)**	**Long COVID (*n* = 105)**	**Statistic**	***P***
Age, M (Q1, Q3)	55.00 (35.00, 71.00)	58.00 (40.00, 69.00)	*Z* = −0.60	0.546
Height, M (Q1, Q3)	165.00 (160.00, 172.00)	165.00 (158.00, 172.00)	*Z* = −0.10	0.918
Weight, M (Q1, Q3)	62.00 (56.00, 73.00)	62.00 (55.00, 70.00)	*Z* = −1.38	0.168
BMI, M (Q1, Q3)	22.89 (21.14, 26.42)	22.52 (20.57, 24.78)	*Z* = −1.92	0.055
**Sex**, ***n*** **(%)**				
Male	54 (51.43)	54 (51.43)	χ^2^ = 0.00	1.000
Female	51 (48.57)	51 (48.57)		
**Number of vaccinations**, ***n*** **(%)**				
One dose	10 (9.52)	10 (9.52)	-	0.456
Two doses	2 (1.90)	5 (4.76)		
Three doses	18 (17.14)	25 (23.81)		
Four or more doses	75 (71.42)	65 (61.91)		
**Number of infections**, ***n*** **(%)**				
Prior infection	33 (31.43)	15 (14.29)	-	**0.008**
Two prior infections	62 (59.05)	68 (64.76)		
Three prior infections	8 (7.62)	17 (16.19)		
More than three prior infections	2 (1.90)	5 (4.76)		
**Clinical classification**, ***n*** **(%)**				
Undiagnosed	5 (4.76)	1 (0.95)	-	0.074
Mild	83 (79.05)	78 (74.29)		
Moderate	14 (13.33)	25 (23.81)		
Severe/critical	3 (2.86)	1 (0.95)		
**Therapy method**, ***n*** **(%)**				
untreated	1 (0.95)	1 (0.95)	χ^2^ = 4.56	0.336
Taking medicine at home	22 (20.95)	12 (11.43)		
Ambulatory treatment	42 (40.00)	40 (38.10)		
Emergency treatment	6 (5.71)	8 (7.62)		
Hospitalization	34 (32.38)	44 (41.90)		
Analgesic-antipyretic, *n* (%)	54 (51.43)	56 (53.33)	χ^2^ = 0.08	0.782
Western medicine for cold *n* (%)	16 (15.24)	29 (27.62)	*xχ*^2^ = 4.78	**0.029**
Cough Medicine, *n* (%)	17 (16.19)	24 (22.86)	χ^2^ = 1.49	0.223
Chinese patent medicine, *n* (%)	22 (20.95)	30 (28.57)	χ^2^ = 1.64	0.201
Chinese herbal decoction, *n* (%)	8 (7.62)	6 (5.71)	χ^2^ = 0.31	0.580
Paxlovid, *n* (%)	2 (1.90)	2 (1.90)	χ^2^ = 0.00	1.000
Azvudine, n (%)	7 (6.67)	2 (1.90)	χ^2^ = 1.86	0.173
Diagnosed with pneumonia, *n* (%)	23 (21.90)	43 (40.95)	χ^2^ = 8.84	**0.003**
Ventilator-assisted treatment, *n* (%)	3 (2.86)	3 (2.86)	χ^2^ = 0.00	1.000
ICU treatment, *n* (%)	1 (0.95)	2 (1.90)	χ^2^ = 0.00	1.000

t, *t*-test; χ^2^, Chi-square test; -, Fisher exact; SD, standard deviation.

**Figure 2:**
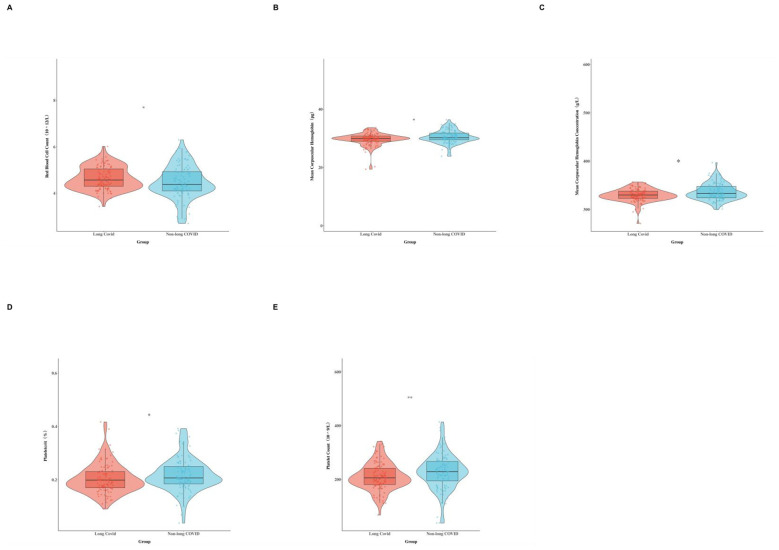
The hematological characteristics of patients with long COVID and non-long COVID after follow-up investigation. **(A)** Red blood cell count. **(B)** Mean corpuscular hemoglobin. **(C)** Mean corpuscular hemoglobin concentration. **(D)** Plateletcrit. **(E)** Platelet count.

Serological results showed that the red blood cell count of long COVID patients was significantly higher than that of the non-long COVID group (*p* = 0.045), while the mean corpuscular hemoglobin (*p* = 0.034), mean corpuscular hemoglobin concentration (*p* = 0.015), plateletcrit (*p* = 0.020) and platelet count (*p* = 0.009) were significantly lower than those of the non-long COVID group. See Figure 2 and Supplementary Table 4.

## 4 Discussion

Long COVID has emerged as a prevalent and persistent public health concern. Previous studies have reported that the incidence of long COVID symptoms among individuals recovering from acute SARS-CoV-2 infection ranges from 10 to 15% (10). A follow-up study of Omicron-infected individuals in Shanghai found that 8.89% continued to experience long COVID symptoms 6 months after clinical recovery (11). In contrast, another longitudinal study with a one-year follow-up period reported that approximately 45% of patients developed long COVID manifestations (4). In the present study, the prevalence of long COVID was 6.52%, which is lower than estimates from most prior investigations. This discrepancy may be attributed to the timing of cohort enrollment, which coincided with the peak of local SARS-CoV-2 cases. At this stage, a substantial proportion of individuals had experienced one or more prior infections, resulting in a degree of pre-existing hybrid immunity. This immune background may have attenuated the severity and likelihood of developing long COVID following reinfection, thereby contributing to the observed lower prevalence.

Nevertheless, our findings confirm that long COVID remains a significant risk even after multiple infections. Common long COVID symptoms include fatigue, cognitive impairment (e.g., memory decline and brain fog), reduced exercise tolerance, and neuropsychiatric disturbances. Consistent with previous reports, the predominant symptoms identified in this cohort were fatigue, cough, expectoration, insomnia, dry or itchy throat, throat pain, and loss of appetite (3, 12). Notably, there remains a paucity of prospective follow-up studies on long COVID within domestic populations, and many existing studies rely on retrospective designs, which are susceptible to recall bias. To address this gap, the current study employed a prospective cohort design, establishing a well-defined cohort of confirmed COVID-19 patients to investigate the incidence, progression systematically, and associated factors of long COVID.

Similar to previous studies, This study identifies age as a key risk factor for long COVID (13, 14). Meanwhile, our study was limited to a 3-month observation period. In contrast, other evidence indicates that a substantial proportion of patients continue to experience persistent symptoms more than two years after SARS-CoV-2 infection—a finding that underscores the potential moderating role of age in long-term symptom trajectory (14). The severity of acute-phase illness is positively associated with long COVID risk: patients experiencing more severe initial symptoms, prolonged hospitalization, or requiring intensive care exhibit a substantially higher likelihood of developing long-term sequelae. Additionally, individuals with certain infection-predisposing chronic conditions face an increased risk of long COVID following SARS-CoV-2 infection. In a survey conducted in the United States, each 10-year increment in age beyond 40 years is significantly associated with an elevated risk of persistent long COVID (15). Older adults are more susceptible to long COVID due to age-related immune dysregulation (e.g., immunosenescence and inflammaging), diminished viral clearance, pre-existing chronic conditions (16, 17), and immunosenescence-related impaired immune cell activity, reduced antigen responsiveness, and persistent low-grade inflammation (inflammaging) (18, 19). These factors hinder SARS-CoV-2 elimination, potentially forming viral reservoirs (e.g., in the gastrointestinal tract), while persistent antigenic stimulation drives chronic inflammation and autoimmune activation to contribute to long COVID pathogenesis (20, 21). Concurrently, the increased burden of comorbidities in older populations may further amplify disease progression and symptom persistence.

Currently, the diagnosis of long COVID primarily relies on self-reported symptoms and exclusionary criteria, lacking objective and specific biomarkers. This limitation not only complicates clinical diagnosis but also impedes the evaluation of therapeutic interventions and the development of targeted pharmacological treatments. In recent years, researchers have actively explored potential biomarkers using advanced technologies such as proteomics, metabolomics, and multi-omics approaches. Several studies have identified candidate markers, including altered immune molecules and metabolic abnormalities (22, 23). However, most of these findings remain in the exploratory phase and have not yet been validated for clinical use. More importantly, many of these high-throughput detection methods are costly and technically demanding, limiting their feasibility for large-scale screening or routine monitoring in clinical settings. Therefore, identifying biomarkers that can objectively reflect disease status, predict prognosis, and be readily implemented in routine clinical practice has become a critical priority in long COVID research. In this study, long COVID patients exhibited a significantly higher red blood cell count compared to non-long COVID individuals, while mean corpuscular hemoglobin (MCH), mean corpuscular hemoglobin concentration (MCHC), plateletcrit, and platelet count were significantly lower. The reduction in MCH and MCHC may indicate underlying anemia, particularly abnormal erythropoiesis resulting from impaired iron utilization. The elevated red blood cell count may reflect a compensatory mechanism to enhance oxygen delivery (24, 25). Previous studies have reported increased morphological abnormalities in red blood cells among long COVID patients, with the degree of abnormality positively correlated with fatigue severity (26). On the other hand, a one-year follow-up study of 214 individuals infected with SARS-CoV-2 found that early-stage disturbances in iron metabolism were significantly associated with the subsequent development of long COVID (8). Several studies have demonstrated that long COVID is associated with iron-related alterations, including disruption of iron homeostasis, impaired erythropoiesis, and immune dysfunction, all of which may collectively contribute to reduced oxygen transport efficiency, inflammatory imbalance, and the persistence of symptoms (8, 27, 28). Similarly, chronic inflammatory milieu may directly or indirectly contribute to erythrocyte dysfunction and structural damage (29). However, some factors (pre-existing iron deficiency, bleeding, menorrhagia) may still exert subtle potential impacts, which were not fully explored here and represent a study limitation. Future research should incorporate these variables to further validate the independent association between long COVID and hematological parameter alterations. Furthermore, at present, there is a lack of direct evidence for iron and related biomarkers. In the future, researchers will need to conduct further investigations.

This study has several limitations. First, this study is limited to a single month and a specific provincial cohort, which may affect the generalizability of long COVID prevalence estimates and lead to potential biases. The epidemic intensity?dominant circulating strain and local targeted prevention, control and standardized diagnosis and treatment policies in the study period are specific to the region, which may differ from those in other areas or epidemic phases, thus influencing the identification and statistical results of long COVID cases. Therefore, the findings are more applicable to the local infected population in the corresponding period, and multi-center, multi-period and multi-regional studies are needed in the future to verify and supplement the prevalence data of long COVID. Second, the impact of viral co-infections was not examined. Currently, respiratory infections are increasingly complex, with overlapping peaks of SARS-CoV-2, influenza virus, and respiratory syncytial virus (RSV) circulation. Evidence indicates that co-infection with SARS-CoV-2 and other respiratory pathogens significantly worsen acute disease severity, increasing risks of pneumonia and respiratory failure (30). Then, in this study, the selection bias of the population and the recall bias could not be avoided. In this study, the selection bias of the population and the recall bias could not be avoided. Finally, in our analysis of hematological indicators, we measured multiple parameters but did not apply multiplicity adjustments (e.g., Bonferroni or false discovery rate correction) during statistical testing, thereby increasing the likelihood of Type I errors. In the future, multi-center and cross-regional studies will be necessary as a key direction for validating our research results in the future, in order to verify our findings in different populations. Future research must urgently investigate the potential role of co-infections in shaping long COVID outcomes. Moreover, for long COVID patients with suspected iron dysregulation, future therapeutic strategies may focus on the precise modulation of hepcidin levels or enhanced intracellular iron mobilization as a hypothetical strategy to improve relevant clinical manifestations—though this strategy requires validation via targeted mechanistic iron metabolism studies.

## Data Availability

The raw data supporting the conclusions of this article will be made available by the authors, withou undue reservation.
