# Supplementary material for: Analysis of Long COVID characteristics and risk factors in individuals infected with COVID-19: a follow-up study based on a cohort of 2,792 participants
Source: Front Public Health. 2026 Mar 10;14:1760355. doi: 10.3389/fpubh.2026.1760355 (PMC13008914; doi:10.3389/fpubh.2026.1760355)

Supplemental instrument 1：Long-term Symptom Baseline survey Questionnaire for COVID-19 Infected Individuals

Part 1: Basic Information

1. Patient's Name: ____________

2. Mobile Phone Number: ________________________

3. Type of Identification Document: □ ID Card □ Passport □ Other ______________

3.1 Document Number: ____________________________

4. Gender: ____________

5. Age: ____________ years old

6. Current Address: ____

7. Ethnicity: ____________

8. Occupation (Occupation in National Notifiable Disease Reporting System): ____________

9. Height: ____________ centimeters (cm)

10. Weight: ____________ kilograms (kg)

11. Number of COVID-19 Vaccine Doses Received? (Single choice, mandatory)

□ 0 doses (skip to Part 2) □ 1 dose □ 2 doses □ 3 doses □ 4 or more doses

Part 2: Initial Onset and Medical Consultation Information

1. Onset Date: ____________ year _____ month _____ day (based on National Notifiable Disease Reporting System)

2. Date of Positive Antigen/Nucleic Acid Test in This Survey: ____________ year _____ month _____ day

3. How Many Times Have You Been Infected with COVID-19?

□ 1 time □ 2 times □ 3 times □ More than 3 times

4. All Symptoms Occurring Within the First Week of Illness (Multiple choices allowed)

□ Fever, Temperature: _______℃, Duration: _______ days

□ Chills □ Fatigue □ Dry and Itchy Throat □Throat pain □ Nausea □ Palpitations □ Loss of appetite□ Generalized body aches □ Myalgia □ Headache □ Vertigo□ Joint Pain □ Ostealgia □ lumbago□ Cough □Expectoration □ Nasal Congestion □ Rhinorrhea □ Tachypnea □ Dyspnea□ Chest Tightness □ Chest Pain □ Hyposmia □ Hypogeusia
□ Delays in response □ Hair Loss □ Anxiety □ Depression □ Insomnia □ Other ___________________

5. Clinical Classification (Refer to the 10th Edition of the Diagnosis and Treatment Protocol for Novel Coronavirus Infection)

□ No Hospital Diagnosis □ Mild Type □ Moderate Type (Common Type) □ Severe Type □ Critical Type

6. Treatment Method

□ No Treatment (if yes, skip to Question 12) □ Taking medicine at home □ Ambulatory treatment(including primary clinics) □ Emergency treatment □ Hospitalization

7. Medication Use

Medication

Taken or Not

Analgesic-antipyretic such as ibuprofen and acetaminophen

□ Yes □ No

Western medicine for cold such as Gan Kang and Contac

□ Yes □ No

Cough Medicine such as compound liquorice tablets and ambroxol

□ Yes □ No

Chinese patent medicines such as Lianhua Qingwen

□ Yes □ No

Chinese herbal decoction

□ Yes □ No

Paxlovid

□ Yes □ No

Azvudine

□ Yes □ No

Others

□ Yes __________ □ No

8. After the initial onset, did you undergo a CT scan and be diagnosed with pneumonia? □ Yes □ No

9. Did you use a ventilator for assisted ventilation? □ Yes □ No

10. Did you receive treatment in the ICU? □ Yes □ No

11. Did you have any other complications? □ Yes (Viral Pneumonia, Bacterial Pneumonia, Fungal Pneumonia, Bloodstream Infection, Secondary Infection, Acute Respiratory Distress Syndrome, Respiratory Failure, Encephalopathy, Hepatic Insufficiency, Renal Insufficiency, Cardiac-Related Diseases, Multisystem Inflammatory Syndrome, Disseminated Intravascular Coagulation, Septic Shock, Pulmonary Fibrosis, Convulsions, Skin Rash, Other __________) □ No

Part 3: Daily Lifestyle and Relevant Information

1. Smoking Status (Situation in the three months before onset)

□ Never smoked

□ Occasionally smoked (more than 4 times a week but less than 1 cigarette per day on average)

□ Regularly smoked (more than 1 cigarette per day but less than 20 cigarettes, accumulated for more than 6 months)

□ Heavily smoked (20 cigarettes or more per day, accumulated for more than 6 months)

2. Drinking Status: □ Yes □ No

3. Do you have an irregular schedule? □ Never □ Occasionally (1-2 times a week) □ Frequently (3-4 times a week) □ Always (more than 5 times a week)

4. What is your average daily sleep duration? □ Less than 7 hours □ 7-8 hours □ More than 8 hours

5. Number of Physical Exercise Sessions per Week: □ Less than 2 times □ 2-4 times □ 5-7 times □ More than 8 times

6. Other High-Risk or Relevant Factors:

____

Part 4: Past Medical History and Basic Conditions

1. Do you have chronic lung disease? □ Yes, (Asthma, Bronchiectasis, Chronic Bronchitis, Emphysema, Chronic Obstructive Pulmonary Disease (COPD), Other __________) □ No

2. Do you have cardio-cerebrovascular disease? □ Yes, (Hypertension, Coronary Heart Disease, Myocardial Infarction, Cardiomyopathy, Ischemic Encephalopathy, Hemorrhagic Encephalopathy, Other __________) □ No

3. Do you have metabolic disease? □ Yes, (Diabetes Mellitus, Hyperlipidemia, Other __________) □ No

4. Do you have chronic kidney disease? □ Yes, (Glomerulonephritis, Pyelonephritis, Nephrotic Syndrome, Chronic Renal Failure, Renal Dialysis, Other __________) □ No

5. Do you have chronic liver disease? □ Yes, (Chronic Hepatitis B, Chronic Hepatitis C, Fatty Liver, Liver Cirrhosis, Other __________) □ No

6. Do you have cancer/tumor? □ Yes, (Leukemia, Malignant Lymphoma, Multiple Myeloma, Lung Cancer, Other __________) □ No

7. Do you have immunodeficiency? □ Yes, (HIV Infection/AIDS, Long-term Use of Corticosteroids or Other Immunosuppressive Drugs, Other __________) □ No

Investigator: ________________________ Survey Date: ______ year ____ month ___ day

Reviewer: ________________________ Review Date: ______ year ____ month ___ day

Supplemental instrument 2：Follow-up Questionnaire on Long-term Symptoms of Novel Coronavirus Infection

Number: ____________

Part 1: Basic Information

1. Patient's Name: ____________

2. Mobile Phone Number: ________________________

3. Type of Identification Document: □ ID Card □ Passport □ Other ______________

3.1 Document Number: ____________________________

Part 2: Long-term Symptoms of COVID-19

1. Since the last survey, do you still have symptoms (specifically those that may be caused by COVID-19)，And the duration is more than two months? If no, skip to the end.
□ Yes □ No

2. Did these symptoms newly appear after being infected with COVID-19?

□ Yes (skip to Question 4) □ No (skip to Question 3)

3. Do you think these symptoms are more severe than before this COVID-19 infection?

□ Yes □ No

4. Symptoms that have persisted since the last survey:

SymptomYes/No
Fever□ Yes □ No； Chills□ Yes □ No；Fatigue□ Yes □ No；Dry and itchy throat □ Yes □ No； Throat pain □ Yes □ No；Nausea□ Yes □ No； Palpitation□ Yes □ No；
Loss of appetite□ Yes □ No； Generalized body aches□ Yes □ No；Myalgia□ Yes □ No；
Headache□ Yes □ No； Vertigo □ Yes □ No； Joint pain □ Yes □ No；
Ostealgia□ Yes □ No； lumbago □ Yes □ No； Cough□ Yes □ No；Expectoration□ Yes □ No； Nasal congestion□ Yes □ No；Rhinorrhea□ Yes □ No； Tachypnea □ Yes □ No；
Dyspnea □ Yes □ No； Chest tightness□ Yes □ No；Chest pain□ Yes □ No； Hyposmia□ Yes □ No； Hypogeusia □ Yes □ No；Delays in response□ Yes □ No； Hair loss□ Yes □

No； Anxiety□ Yes □ No；Depression□ Yes □ No； Insomnia□ Yes □ No； Other 1□ Yes □ No； ____________
Other 2□ Yes □ No____________

Note: Depression Symptoms: Characterized by persistent and long-term low mood; Anxiety Symptoms: An unpleasant and complex emotional state of tension, restlessness, worry, and distress caused by an individual's anticipation of an impending possible danger or threat.

Investigator: _______________________ Survey Date: ______ year ____ month ___ day

Reviewer: _______________________ Review Date: ______ year ____ month ___ day

Supplemental Table 1 Living habits and chronic diseases characteristic in participant

Variables	Non Long Covid (n = 2610)	Long Covid (n = 182)	Statistic	*P*
Smoke, n (%)			χ^2=0.29	0.961
Never	2218 (84.98)	155 (85.16)		
Occasionally	203 (7.78)	15 (8.24)		
Often	165 (6.32)	10 (5.49)		
Heavily smoked	24 (0.92)	2 (1.10)		
Drink, n (%)			χ^2=1.93	0.165
No	2235 (85.63)	149 (81.87)		
Yes	375 (14.37)	33 (18.13)		
Irregular Sleep, n (%)			χ^2=3.49	0.322
Never	1480 (56.70)	96 (52.75)		
1-2 times a week	819 (31.38)	58 (31.87)		
3-4 times a week	218 (8.35)	17 (9.34)		
More than five times a week	93 (3.56)	11 (6.04)		
Sleep Time, n (%)			χ^2=12.33	**0.002**
Less than 7 hours	833 (31.92)	81 (44.51)		
7-8 hours	1384 (53.03)	80 (43.96)		
More than 8 hours	393 (15.06)	21 (11.54)		
Frequency Exercise, n (%)			χ^2=2.25	0.522
Less than twice	1697 (65.02)	119 (65.38)		
2-4 times	584 (22.38)	38 (20.88)		
5-7 times	302 (11.57)	21 (11.54)		
More than 8 times	27 (1.03)	4 (2.20)		
Chronic Lung, n (%)			χ^2=36.82	**<.001**
No	2398 (91.88)	143 (78.57)		
Yes	212 (8.12)	39 (21.43)		
Cardiovascular Disease, n (%)			χ^2=23.20	**<.001**
No	2028 (77.70)	113 (62.09)		
Yes	582 (22.30)	69 (37.91)		
Metabolic Disease, n (%)			χ^2=6.66	**0.010**
No	2396 (91.80)	157 (86.26)		

Variables	Non Long Covid (n = 2610)	Long Covid (n = 182)	Statistic	*P*
Yes	214 (8.20)	25 (13.74)		
Chronic Renal, n (%)			χ^2=0.18	0.671
No	2558 (98.01)	177 (97.25)		
Yes	52 (1.99)	5 (2.75)		
Chronic Liver, n (%)			χ^2=9.18	**0.002**
No	2569 (98.43)	173 (95.05)		
Yes	41 (1.57)	9 (4.95)		
Cancer, n (%)			χ^2=0.01	0.914
No	2520 (96.55)	176 (96.70)		
Yes	90 (3.45)	6 (3.30)		
Immunede ficiency, n (%)			χ^2=0.22	0.643
No	2596 (99.46)	180 (98.90)		
Yes	14 (0.54)	2 (1.10)		
Chronic disease, n (%)			-	**<.001**
0	1773 (67.93)	90 (49.45)		
1	543 (20.80)	49 (26.92)		
2	232 (8.89)	28 (15.38)		
3	51 (1.95)	11 (6.04)		
4	10 (0.38)	3 (1.65)		
5	1 (0.04)	1 (0.55)		

Z: Mann-Whitney test, χ^2: Chi-square test, -: Fisher exact

M: Median, Q_1: 1st Quartile, Q_3: 3st Quartile

Supplemental Table 2 The matching pre-post statistical analysis table

Variable	Before PSM						After PSM					
	Total (n = 2792)	Non Long Covid (n = 2610)	Long Covid(n = 182)	Statistic	*P*	SMD	Total (n = 873)	Non Long Covid (n = 695)	Non Long Covid (n = 178)	Statistic	*P*	SMD
BMI, M (Q_1, Q_3)	22.55 (20.38, 24.69)	22.49 (20.31, 24.69)	23.12 (21.02, 24.91)	Z=-2.046	**0.041**	0.108	22.86 (20.76, 25.01)	22.86 (20.73, 25.06)	23.03 (21.01, 24.88)	Z=-0.297	0.766	-0.028
Gender, n (%)				χ^2=1.299	0.254					χ^2=0.071	0.790	
Male	1176 (42.12)	1092 (41.84)	84 (46.15)			0.087	405 (46.39)	324 (46.62)	81 (45.51)			-0.022
Female	1616 (57.88)	1518 (58.16)	98 (53.85)			-0.087	468 (53.61)	371 (53.38)	97 (54.49)			0.022
Number Vaccine, n (%)				χ^2=2.803	0.423					χ^2=2.085	0.555	
One dose	305 (10.92)	282 (10.80)	23 (12.64)			0.055	99 (11.34)	77 (11.08)	22 (12.36)			0.039
Two doses	88 (3.15)	79 (3.03)	9 (4.95)			0.088	27 (3.09)	19 (2.73)	8 (4.49)			0.085
Three doses	534 (19.13)	500 (19.16)	34 (18.68)			-0.012	159 (18.21)	125 (17.99)	34 (19.10)			0.028
Four or more doses	1865 (66.8)	1749 (67.01)	116 (63.74)			-0.068	588 (67.35)	474 (68.20)	114 (64.04)			-0.087
Number of Infection, n (%)				χ^2=10.413	**0.015**					χ^2=0.135	0.987	

Variable	Before PSM						After PSM					
	Total (n = 2792)	Non Long Covid (n = 2610)	Long Covid(n = 182)	Statistic	*P*	SMD	Total (n = 873)	Non Long Covid (n = 695)	Non Long Covid (n = 178)	Statistic	*P*	SMD
Prior infection	616 (22.06)	590 (22.61)	26 (14.29)			-0.238	124 (14.2)	98 (14.10)	26 (14.61)			0.014
Two prior infections	1769 (63.36)	1648 (63.14)	121 (66.48)			0.071	587 (67.24)	469 (67.48)	118 (66.29)			-0.025
Three prior infections	333 (11.93)	307 (11.76)	26 (14.29)			0.072	126 (14.43)	100 (14.39)	26 (14.61)			0.006
More than three prior infections	74 (2.65)	65 (2.49)	9 (4.95)			0.113	36 (4.12)	28 (4.03)	8 (4.49)			0.022
Smoke, n (%)				χ^2=0.292	0.961					χ^2=2.245	0.523	
Never	2373 (84.99)	2218 (84.98)	155 (85.16)			0.005	717 (82.13)	565 (81.29)	152 (85.39)			0.116
Occasionally	218 (7.81)	203 (7.78)	15 (8.24)			0.017	82 (9.39)	67 (9.64)	15 (8.43)			-0.044
Often	175 (6.27)	165 (6.32)	10 (5.49)			-0.036	65 (7.45)	56 (8.06)	9 (5.06)			-0.137
Heavily smoked	26 (0.93)	24 (0.92)	2 (1.10)			0.017	9 (1.03)	7 (1.01)	2 (1.12)			0.011
Drink, n (%)				χ^2=1.932	0.165					χ^2=0.094	0.759	
No	2384 (85.39)	2235 (85.63)	149 (81.87)			-0.098	718 (82.25)	573 (82.45)	145 (81.46)			-0.025
Yes	408 (14.61)	375 (14.37)	33 (18.13)			0.098	155 (17.75)	122 (17.55)	33 (18.54)			0.025
Irregular Sleep, n (%)				χ^2=3.493	0.322					χ^2=0.303	0.959	

Variable	Before PSM						After PSM					
	Total (n = 2792)	Non Long Covid (n = 2610)	Long Covid(n = 182)	Statistic	*P*	SMD	Total (n = 873)	Non Long Covid (n = 695)	Non Long Covid (n = 178)	Statistic	*P*	SMD
Never	1576 (56.45)	1480 (56.70)	96 (52.75)			-0.079	450 (51.55)	355 (51.08)	95 (53.37)			0.046
1-2 times a week	877 (31.41)	819 (31.38)	58 (31.87)			0.010	291 (33.33)	234 (33.67)	57 (32.02)			-0.035
3-4 times a week	235 (8.42)	218 (8.35)	17 (9.34)			0.034	82 (9.39)	66 (9.50)	16 (8.99)			-0.018
More than five times a week	104 (3.72)	93 (3.56)	11 (6.04)			0.104	50 (5.73)	40 (5.76)	10 (5.62)			-0.006
Sleep Time, n (%)				χ^2=12.328	**0.002**					χ^2=0.298	0.862	
Less than 7 hours	914 (32.74)	833 (31.92)	81 (44.51)			0.253	367 (42.04)	290 (41.73)	77 (43.26)			0.031
7-8 hours	1464 (52.44)	1384 (53.03)	80 (43.96)			-0.183	408 (46.74)	328 (47.19)	80 (44.94)			-0.045
More than 8 hours	414 (14.83)	393 (15.06)	21 (11.54)			-0.110	98 (11.23)	77 (11.08)	21 (11.80)			0.022
Frequency of Exercise, n (%)				χ^2=2.248	0.522					χ^2=0.306	0.959	
Less than twice	1816 (65.04)	1697 (65.02)	119 (65.38)			0.008	577 (66.09)	461 (66.33)	116 (65.17)			-0.024
2-4 times	622 (22.28)	584 (22.38)	38 (20.88)			-0.037	180 (20.62)	142 (20.43)	38 (21.35)			0.022
5-7 times	323 (11.57)	302 (11.57)	21 (11.54)			-0.001	100 (11.45)	80 (11.51)	20 (11.24)			-0.009
More than 8 times	31 (1.11)	27 (1.03)	4 (2.20)			0.079	16 (1.83)	12 (1.73)	4 (2.25)			0.035
Chronic Lung, n (%)				χ^2=36.816	**<.001**					χ^2=0.266	0.606	

Variable	Before PSM						After PSM					
	Total (n = 2792)	Non Long Covid (n = 2610)	Long Covid(n = 182)	Statistic	*P*	SMD	Total (n = 873)	Non Long Covid (n = 695)	Non Long Covid (n = 178)	Statistic	*P*	SMD
No	2541 (91.01)	2398 (91.88)	143 (78.57)			-0.324	713 (81.67)	570 (82.01)	143 (80.34)			-0.042
Yes	251 (8.99)	212 (8.12)	39 (21.43)			0.324	160 (18.33)	125 (17.99)	35 (19.66)			0.042
Cardiovascular Disease, n (%)				χ^2=23.196	**<.001**					χ^2=0.510	0.475	
No	2141 (76.68)	2028 (77.70)	113 (62.09)			-0.322	574 (65.75)	461 (66.33)	113 (63.48)			-0.059
Yes	651 (23.32)	582 (22.30)	69 (37.91)			0.322	299 (34.25)	234 (33.67)	65 (36.52)			0.059
Metabolic Disease, n (%)				χ^2=6.664	**0.010**					χ^2=0.096	0.757	
No	2553 (91.44)	2396 (91.80)	157 (86.26)			-0.161	754 (86.37)	599 (86.19)	155 (87.08)			0.027
Yes	239 (8.56)	214 (8.20)	25 (13.74)			0.161	119 (13.63)	96 (13.81)	23 (12.92)			-0.027
Chronic Renal, n (%)				χ^2=0.181	0.671					χ^2=0.305	0.580	
No	2735 (97.96)	2558 (98.01)	177 (97.25)			-0.046	848 (97.14)	674 (96.98)	174 (97.75)			0.052
Yes	57 (2.04)	52 (1.99)	5 (2.75)			0.046	25 (2.86)	21 (3.02)	4 (2.25)			-0.052
Chronic Liver, n (%)				χ^2=9.179	**0.002**					χ^2=0.022	0.882	
No	2742	2569 (98.43)	173 (95.05)			-0.156	847 (97.02)	674 (96.98)	173 (97.19)			0.013

Variable	Before PSM						After PSM					
	Total (n = 2792)	Non Long Covid (n = 2610)	Long Covid(n = 182)	Statistic	*P*	SMD	Total (n = 873)	Non Long Covid (n = 695)	Non Long Covid (n = 178)	Statistic	*P*	SMD
	(98.21)											
Yes	50 (1.79)	41 (1.57)	9 (4.95)			0.156	26 (2.98)	21 (3.02)	5 (2.81)			-0.013
Cancer, n (%)				χ^2=0.012	0.914					χ^2=0.114	0.735	
No	2696 (96.56)	2520 (96.55)	176 (96.70)			0.008	845 (96.79)	672 (96.69)	173 (97.19)			0.030
Yes	96 (3.44)	90 (3.45)	6 (3.30)			-0.008	28 (3.21)	23 (3.31)	5 (2.81)			-0.030
Immune deficiency, n (%)				χ^2=0.215	0.643					χ^2=0.286	0.593	
No	2776 (99.43)	2596 (99.46)	180 (98.90)			-0.054	868 (99.43)	692 (99.57)	176 (98.88)			-0.066
Yes	16 (0.57)	14 (0.54)	2 (1.10)			0.054	5 (0.57)	3 (0.43)	2 (1.12)			0.066

Supplemental Table 3 The information summary table of participant after PSM

Variables	Non Long Covid (n = 695)	Long Covid (n = 178)	Statistic	*P*
Age, M (Q_1, Q_3)	56.00 (33.00, 71.00)	59.00 (43.25, 72.00)	Z=-2.56	**0.010**
Fever, n(%)			χ^2=0.79	0.376
No	154 (22.16)	45 (25.28)		
Yes	541 (77.84)	133 (74.72)		
Chilly, n(%)			χ^2=0.48	0.486
No	559 (80.43)	139 (78.09)		
Yes	136 (19.57)	39 (21.91)		
Fatigue, n(%)			χ^2=2.74	0.098
No	460 (66.19)	106 (59.55)		
Yes	235 (33.81)	72 (40.45)		
Dry and itchy throat, n(%)			χ^2=1.13	0.288
No	578 (83.17)	142 (79.78)		
Yes	117 (16.83)	36 (20.22)		
Throat pain, n(%)			χ^2=0.04	0.848
No	466 (67.05)	118 (66.29)		
Yes	229 (32.95)	60 (33.71)		
Nausea, n(%)			χ^2=0.58	0.446
No	667 (95.97)	173 (97.19)		
Yes	28 (4.03)	5 (2.81)		
Palpitation, n(%)			χ^2=0.02	0.886
No	662 (95.25)	170 (95.51)		
Yes	33 (4.75)	8 (4.49)		
Loss of appetite, n(%)			χ^2=1.15	0.284
No	614 (88.35)	152 (85.39)		
Yes	81 (11.65)	26 (14.61)		
Generalized body aches, n(%)			χ^2=7.80	**0.005**
No	559 (80.43)	126 (70.79)		
Yes	136 (19.57)	52 (29.21)		
Myalgia, n(%)			χ^2=3.33	0.068

Variables	Non Long Covid (n = 695)	Long Covid (n = 178)	Statistic	*P*
No	583 (83.88)	139 (78.09)		
Yes	112 (16.12)	39 (21.91)		
Headache, n(%)			χ^2=0.16	0.692
No	569 (81.87)	148 (83.15)		
Yes	126 (18.13)	30 (16.85)		
Vertigo, n(%)			χ^2=0.08	0.778
No	603 (86.76)	153 (85.96)		
Yes	92 (13.24)	25 (14.04)		
Joint pain, n(%)			χ^2=0.84	0.358
No	650 (93.53)	163 (91.57)		
Yes	45 (6.47)	15 (8.43)		
Ostealgia, n(%)			χ^2=0.64	0.425
No	681 (97.99)	172 (96.63)		
Yes	14 (2.01)	6 (3.37)		
Lumbago, n(%)			χ^2=0.18	0.670
No	665 (95.68)	169 (94.94)		
Yes	30 (4.32)	9 (5.06)		
Cough, n(%)			χ^2=0.00	0.962
No	345 (49.64)	88 (49.44)		
Yes	350 (50.36)	90 (50.56)		
Expectoration, n(%)			χ^2=1.53	0.217
No	503 (72.37)	137 (76.97)		
Yes	192 (27.63)	41 (23.03)		
Nasal congestion, n(%)			χ^2=0.35	0.554
No	589 (84.75)	154 (86.52)		
Yes	106 (15.25)	24 (13.48)		
Rhinorrhea, n(%)			χ^2=2.69	0.101
No	556 (80.00)	152 (85.39)		
Yes	139 (20.00)	26 (14.61)		
Tachypnea, n(%)			χ^2=3.36	0.067
No	664 (95.54)	164 (92.13)		
Yes	31 (4.46)	14 (7.87)		
Dyspnea, n(%)			χ^2=3.02	0.082

Variables	Non Long Covid (n = 695)	Long Covid (n = 178)	Statistic	*P*
No	660 (94.96)	163 (91.57)		
Yes	35 (5.04)	15 (8.43)		
Chest tightness, n(%)			χ^2=0.16	0.691
No	624 (89.78)	158 (88.76)		
Yes	71 (10.22)	20 (11.24)		
Chest pain, n(%)			χ^2=0.70	0.401
No	676 (97.27)	171 (96.07)		
Yes	19 (2.73)	7 (3.93)		
Hyposmia, n(%)			χ^2=0.12	0.734
No	664 (95.54)	169 (94.94)		
Yes	31 (4.46)	9 (5.06)		
Hypogeusia, n(%)			χ^2=11.53	**<.001**
No	666 (95.83)	159 (89.33)		
Yes	29 (4.17)	19 (10.67)		
Delays in response, n(%)			χ^2=0.00	0.994
No	678 (97.55)	173 (97.19)		
Yes	17 (2.45)	5 (2.81)		
Hair loss, n(%)			χ^2=0.00	1.000
No	687 (98.85)	176 (98.88)		
Yes	8 (1.15)	2 (1.12)		
Anxiety, n(%)			χ^2=0.58	0.447
No	687 (98.85)	174 (97.75)		
Yes	8 (1.15)	4 (2.25)		
Depression, n(%)			χ^2=0.08	0.778
No	691 (99.42)	176 (98.88)		
Yes	4 (0.58)	2 (1.12)		
Insomnia, n(%)			χ^2=0.49	0.483
No	668 (96.12)	169 (94.94)		
Yes	27 (3.88)	9 (5.06)		
Clinical classification, n(%)			χ^2=8.13	**0.043**
Undiagnosed	31 (4.46)	3 (1.69)		
Mild	557 (80.14)	134 (75.28)		
Moderate	93 (13.38)	36 (20.22)		

Variables	Non Long Covid (n = 695)	Long Covid (n = 178)	Statistic	*P*
Severe/Critical	14 (2.01)	5 (2.81)		
Therapy method, n(%)			χ^2=3.79	0.435
Untreated	20 (2.88)	1 (0.56)		
Taking medicine at home	75 (10.79)	18 (10.11)		
Ambulatory treatment	304 (43.74)	80 (44.94)		
Emergency treatment	46 (6.62)	10 (5.62)		
Hospitalization	250 (35.97)	69 (38.76)		
Analgesicantipyretic, n(%)			χ^2=0.04	0.851
No	342 (49.21)	89 (50.00)		
Yes	353 (50.79)	89 (50.00)		
Western medicine for colds, n(%)			χ^2=0.24	0.627
No	539 (77.55)	135 (75.84)		
Yes	156 (22.45)	43 (24.16)		
Cough Medicine, n(%)			χ^2=3.21	0.073
No	565 (81.29)	134 (75.28)		
Yes	130 (18.71)	44 (24.72)		
Chinese patent medicine, n(%)			χ^2=0.92	0.337
No	490 (70.50)	132 (74.16)		
Yes	205 (29.50)	46 (25.84)		
Chinese herbaldecoction, n(%)			χ^2=2.74	0.098
No	671 (96.55)	167 (93.82)		
Yes	24 (3.45)	11 (6.18)		
Paxlovid, n(%)			χ^2=0.05	0.832
No	670 (96.40)	171 (96.07)		
Yes	25 (3.60)	7 (3.93)		
Azvudine, n(%)			χ^2=0.21	0.649
No	676 (97.27)	172 (96.63)		
Yes	19 (2.73)	6 (3.37)		
Diagnosed with			χ^2=15.95	**<.001**

Variables	Non Long Covid (n = 695)	Long Covid (n = 178)	Statistic	*P*
pneumonia, n(%)				
No	529 (76.12)	109 (61.24)		
Yes	166 (23.88)	69 (38.76)		
Received ventilator assistedtreatment, n(%)			χ^2=0.00	1.000
No	678 (97.55)	174 (97.75)		
Yes	17 (2.45)	4 (2.25)		
Be admitted to the ICU for treatment, n(%)			χ^2=0.00	1.000
No	685 (98.56)	176 (98.88)		
Yes	10 (1.44)	2 (1.12)		
Complications during the acute phase of covid19, n(%)			χ^2=2.31	0.129
No	648 (93.24)	160 (89.89)		
Yes	47 (6.76)	18 (10.11)		

Supplemental Table 4 The results of the hematology tests in the case and control group.

**Variables**	**Total (n = 210)**	**Non long Covid (n = 105)**	**Long Covid(n = 105)**	**Statistic**	**P**
Platelet Count （10＾9/L）, M (Q_1, Q_3)	216.00 (182.00, 252.00)	229.00 (195.00, 267.00)	206.00 (181.00, 242.00)	Z=-2.62	0.009
Mean Corpuscular Hemoglobin Concentration（g/L）, M (Q_1, Q_3)	331.00 (323.00, 341.00)	333.00 (324.00, 348.00)	330.00 (323.00, 338.00)	Z=-2.42	0.015
Plateletcrit（%）, M (Q_1, Q_3)	0.20 (0.17, 0.24)	0.21 (0.18, 0.25)	0.20 (0.17, 0.23)	Z=-2.33	0.020
Mean Corpuscular Hemoglobin （pg）, M (Q_1, Q_3)	30.00 (29.20,	30.20 (29.50,	30.00 (29.00,	Z=-2.12	0.034

	31.30)	31.80)	30.80)		
Red Blood Cell Count（10＾12/L）, M (Q_1, Q_3)	4.48 (4.17, 5.00)	4.38 (4.10, 4.93)	4.57 (4.30, 5.05)	Z=-2.00	0.045
Lymphocyte（10＾9/L）, M (Q_1, Q_3)	1.87 (1.51, 2.30)	1.96 (1.53, 2.38)	1.80 (1.46, 2.13)	Z=-1.56	0.119
Hematocrit（%）, M (Q_1, Q_3)	40.90 (37.58, 45.02)	40.50 (37.00, 44.30)	41.55 (38.20, 45.18)	Z=-1.40	0.162
Lymphocyte Percentage（%）, M (Q_1, Q_3)	32.50 (25.40, 38.40)	34.30 (25.85, 39.40)	31.90 (25.20, 38.10)	Z=-1.26	0.209
Platelet Distribution Width(%), M (Q_1, Q_3)	15.19 (15.15, 15.54)	15.20 (15.16, 15.49)	15.18 (15.14, 15.55)	Z=-1.15	0.248
Eosinophil Percentage（%）, M (Q_1, Q_3)	1.70 (1.00, 2.60)	1.60 (1.00, 2.80)	1.75 (1.08, 2.47)	Z=-0.88	0.381
Basophil percentage（%）, M (Q_1, Q_3)	0.17 (0.10, 0.30)	0.10 (0.10, 0.20)	0.20 (0.10, 0.30)	Z=-0.86	0.388
Hemoglobin（g/L）, M (Q_1, Q_3)	135.00 (127.00, 148.75)	134.00 (126.00, 149.00)	137.00 (129.00, 147.00)	Z=-0.83	0.406
Total Bilirubin（umol/L）, M (Q_1, Q_3)	12.32 (9.54, 15.47)	12.10 (9.30, 15.30)	12.60 (9.90, 16.55)	Z=-0.74	0.458
Aspartate Aminotransferase（U/L）, M (Q_1, Q_3)	21.00 (17.92, 27.00)	21.00 (18.00, 26.30)	21.40 (17.90, 27.00)	Z=-0.73	0.467
Alanine Aminotransferase（U/L）, M (Q_1, Q_3)	18.15 (13.83, 29.00)	19.00 (14.00, 29.71)	17.70 (13.70, 27.40)	Z=-0.64	0.524
White blood cells（10＾9/L）, M (Q_1, Q_3)	5.80 (4.87, 7.08)	5.93 (4.89, 7.26)	5.80 (4.74, 6.83)	Z=-0.60	0.551
Neutrophilic granulocyte percentage（%）, M (Q_1, Q_3)	57.40 (51.40, 64.50)	56.85 (50.68, 64.72)	58.20 (51.90, 64.40)	Z=-0.59	0.552
AST/ALT Ratio, M (Q_1, Q_3)	1.11 (0.81,	1.12 (0.81,	1.11 (0.87,	Z=-0.5	0.583

	1.52)	1.49)	1.53)	5	
Albumin（g/L）, M (Q_1, Q_3)	46.70 (44.40, 49.30)	46.60 (44.18, 49.18)	47.00 (44.80, 49.35)	Z=-0.49	0.628
Monocyte Percentage（%）, M (Q_1, Q_3)	6.60 (5.20, 8.00)	6.60 (5.40, 8.00)	6.55 (5.10, 7.93)	Z=-0.47	0.642
Monocyte（10ˆ9/L）, M (Q_1, Q_3)	0.39 (0.30, 0.50)	0.40 (0.30, 0.50)	0.39 (0.29, 0.50)	Z=-0.38	0.703
Red Cell Distribution Width（%）, M (Q_1, Q_3)	13.10 (12.43, 13.60)	13.00 (12.50, 13.70)	13.10 (12.20, 13.60)	Z=-0.36	0.720
Albumin/Globulin Ratio, M (Q_1, Q_3)	1.76 (1.54, 2.00)	1.76 (1.50, 2.00)	1.74 (1.55, 2.04)	Z=-0.36	0.722
Uric Acid（umol/L）, M (Q_1, Q_3)	305.00 (248.00, 367.30)	293.95 (243.03, 369.68)	312.00 (249.00, 367.10)	Z=-0.22	0.830
Globulin（g/L）, M (Q_1, Q_3)	26.60 (23.35, 29.98)	26.25 (23.30, 30.07)	27.00 (23.72, 29.88)	Z=-0.20	0.842
Creatinine（umol/L）, M (Q_1, Q_3)	64.55 (51.05, 77.75)	62.70 (52.60, 75.90)	67.90 (50.80, 81.60)	Z=-0.15	0.882
Mean Platelet Volume（fL）, M (Q_1, Q_3)	9.60 (8.70, 10.70)	9.60 (8.70, 10.60)	9.50 (8.50, 11.10)	Z=-0.10	0.919
Neutrophile granulocyte（10ˆ9/L）, M (Q_1, Q_3)	3.28 (2.61, 4.29)	3.31 (2.54, 4.38)	3.27 (2.62, 4.17)	Z=-0.09	0.924
Mean Corpuscular Volume（fL）, M (Q_1, Q_3)	90.60 (87.93, 93.52)	90.60 (87.40, 94.20)	90.60 (88.30, 93.20)	Z=-0.06	0.951
Total Protein（g/L）, M (Q_1, Q_3)	73.10 (69.50, 77.00)	72.80 (69.50, 76.82)	73.45 (69.62, 77.00)	Z=-0.04	0.970

Supplemental **Figure 1 Flowchart of Quality Control Standards and Processes for Collected Survey Questionnaires**

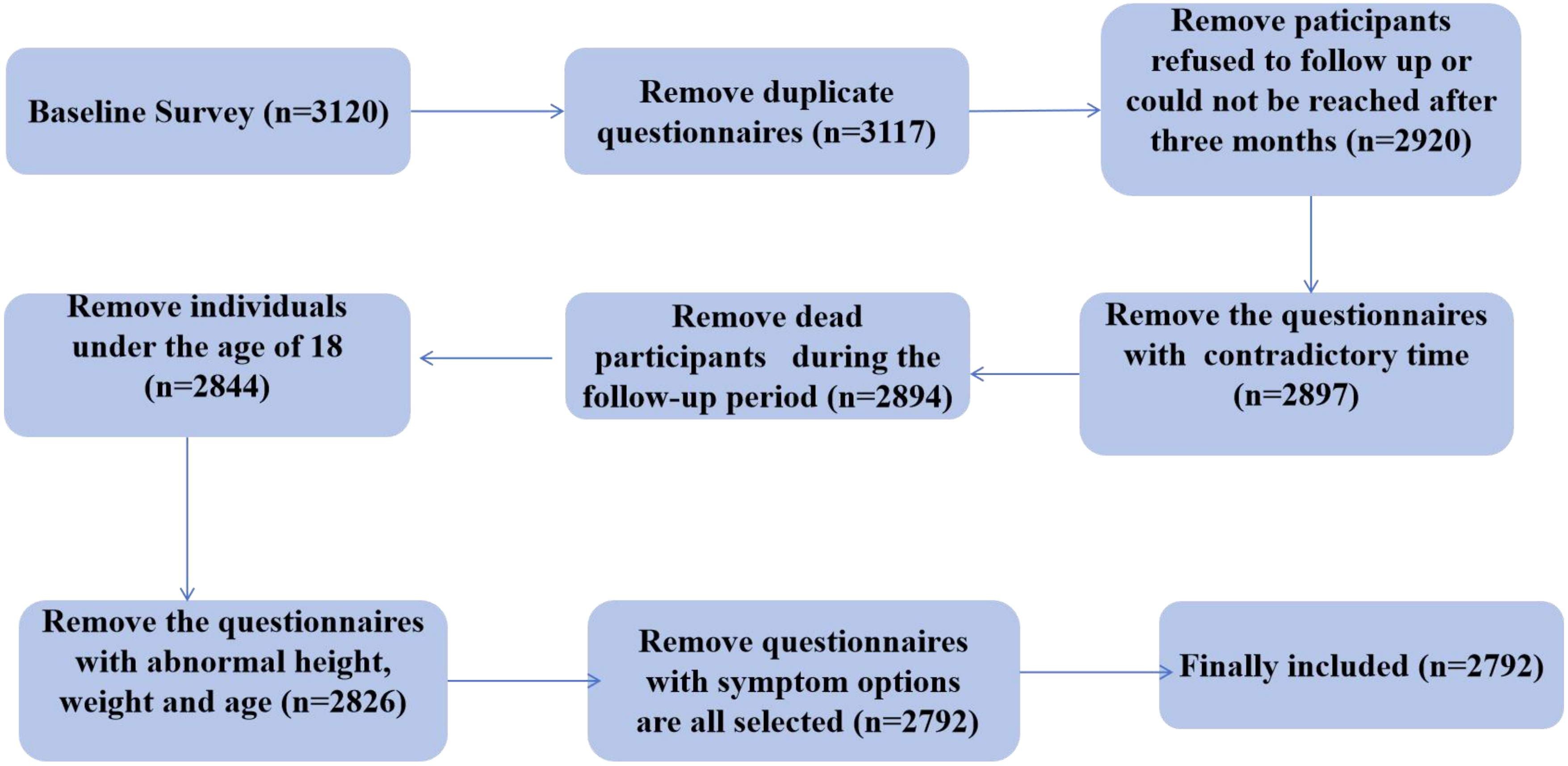

Supplement: Supplementary file 1 [file Data_Sheet_1.pdf]
